# Henoch-Schönlein Purpura: A Case Report and Review of Literature

**DOI:** 10.7759/cureus.69511

**Published:** 2024-09-16

**Authors:** Reem A AlQusaimi

**Affiliations:** 1 Dermatopathology, Al-Amiri Hospital, Kuwait City, KWT

**Keywords:** hsp, immunosuppression, multisystem, purpura, small-vessel vasculitis

## Abstract

Henoch-Schönlein purpura (HSP), or anaphylactoid purpura, is an acute, self-limited small-vessel vasculitis commonly affecting children aged 3-10. It features a classic tetrad: palpable non-thrombocytopenic purpura, abdominal pain, polyarthralgia, and renal involvement. While the prognosis is generally favorable, long-term outcomes depend on renal involvement.

This report describes a two-year-old boy with a two-week history of erythematous purpura, initially on the lower extremities and spreading to other areas, accompanied by painful, pruritic rash, arthralgia, and edema. A clinical diagnosis of HSP was made, and the patient was treated with topical dexpanthenol and oral paracetamol.

This case highlights the importance of recognizing HSP, understanding its manifestations, and guiding treatment and long-term management. Further research is needed to clarify aspects of its pathogenesis, treatment, and prognosis.

## Introduction

Henoch-Schönlein purpura (HSP) is an acute, self-limiting systemic vasculitis affecting small blood vessels, including arterioles, capillaries, and venules. This condition, driven by immune complex deposition of IgA1 in vessel walls and the renal mesangium, typically resolves on its own in about four weeks, though the duration can vary. The primary symptoms include palpable purpura that is not associated with thrombocytopenia, diffuse abdominal pain, polyarthralgia, and renal involvement. Other organ systems affected include the lungs and brain [1].

HSP is the most frequent childhood vasculitis, occurring in 10-20 children per 100,000 annually, predominantly in those aged 3-10, with an average onset at six years. It shows a male predominance of about two-to-one and is more commonly seen in Caucasian children, with African American children rarely affected. The condition tends to be more prevalent in the winter and autumn months, reaching its peak between January and March [2].

This report delineates the case of a two-year-old boy, previously healthy, presenting with a two-week history of palpable erythematous purpura. After further assessment and dermatology consultation, a clinical diagnosis of HSP was established.

## Case presentation

**Figure 1:**
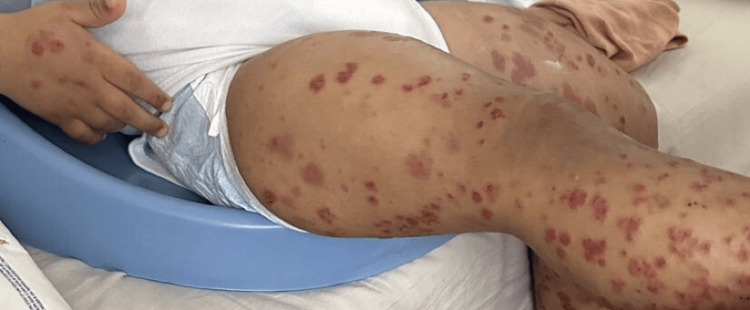
Palpable purpura on the upper and lower extremities

**Figure 2:**
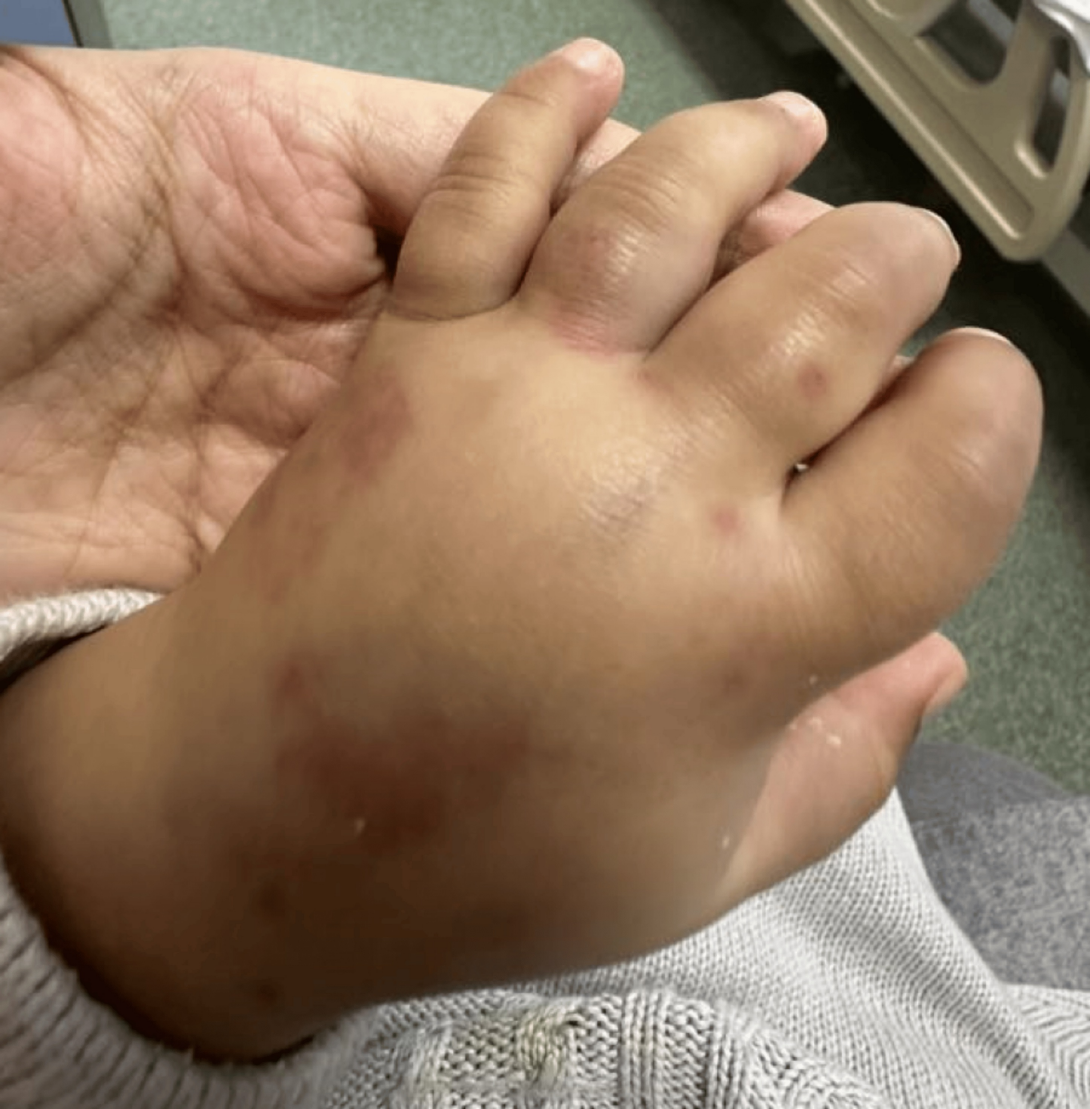
Local edema of the dorsum of the hand

**Figure 3:**
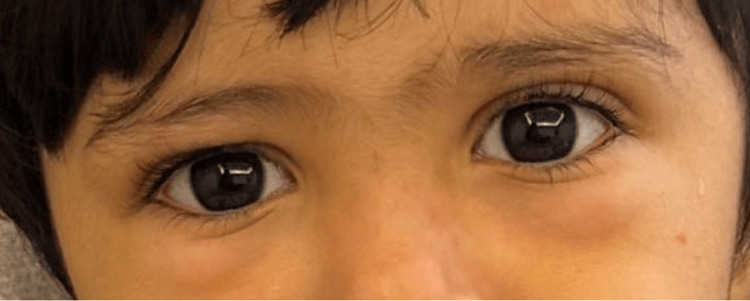
Bilateral periorbital edema

A two-year-old, previously healthy boy presented to the pediatrics emergency department complaining of a two-week history of arthralgia and palpable purpura. The purpura was initially seen on the lower extremities, spreading to the trunk and upper extremities. The mother also noticed increasing bilateral ankle swelling and swelling around the eyes that waxes and wanes. At that time, there were no signs of mucosal involvement or conjunctival or genital lesions. There were no complaints of fever, fatigability, hematuria, abdominal pain, or orchitis. The mother also denied any recent viral illness, novel medication intake, contact with a sick person, and a history of similar attacks. On examination, the patient was vitally stable and afebrile. He was conscious and alert. Widespread palpable purpura was seen, however, primarily on the lower limbs, trunk, and upper limbs (Figure 1). The rash was associated with tenderness. Edema was also appreciated, predominantly on the dorsum of the hands, ankles, and periorbitals (Figure 2 and Figure 3). There were no signs of dehydration, lymphadenopathy conjunctivitis, or orchitis, as both testes were palpable and non-tender. 

**Figure 4:**
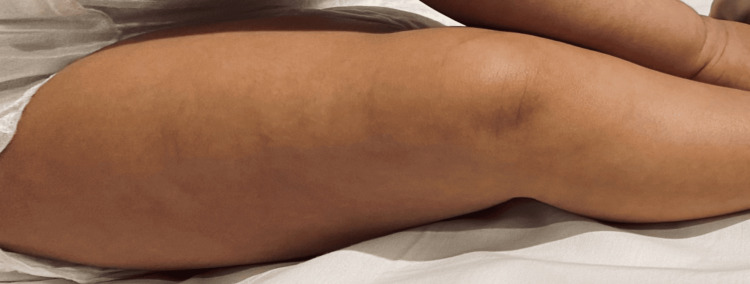
Follow-up after four weeks

After close assessment and thorough history-taking, a diagnosis of HSP was established, and hence, the patient was admitted to the general ward to be kept under close observation and to receive the appropriate supportive management. Thereafter, routine blood workup, including complete blood count and blood film, renal, liver, and coagulation profiles, C-reactive protein, erythrocyte sedimentation rate, antistreptolysin O titers, and urinalysis, were ordered, all of which appeared to be within the normal range, with a minimal elevation of the C-reactive protein and erythrocyte sedimentation rate (Table 1 and Table 2). The patient was initially treated conservatively with intravenous fluid and oral paracetamol when needed. He was then seen by a dermatologist in the ward, who advised adding topical dexpanthenol cream to the initial treatment plan after diagnosing the patient with HSP. After staying in the ward for three days, the patient was discharged on paracetamol. After four weeks of follow-up, the skin lesions disappeared and the edema subsided and the mother was satisfied with the results (Figure 4). 

**Table 1:** Routine blood workup upon admission WBC: white blood cell; INR: international normalized ratio; CRP: C-reactive protein; ESR: erythrocyte sedimentation rate

Test	Value	Reference range
WBC	9	4-10 (10^9^/L)
Hemoglobin	135	130-170 (g/L)
Creatinine	29	21-65 (umol/L)
Sodium	135	134-144 (mmol/L)
Potassium	5.04	3.6-5.1 (mmol/L)
Total protein	57	57-80 (g/L)
Albumin	35	35-52 (g/L)
INR	1.13	1-2
CRP	22.3	0-5 (mg/L)
ESR	28	0-15 (mm/hr)

**Table 2:** Urinalysis upon admission WBC: white blood cell; RBC: red blood cell

Test	Results
Ph	7.5
Glucose	Negative
Ketone	Negative
Protein	Negative
Bilirubin	Negative
Urobilinogen	3.2 (umol/L)
Specific gravity	1.009
Nitrite	Negative
Blood	Negative
Leukocyte	Negative
Clarity	Clear
Color	Yellow
WBC	0-1
RBC	Nil

## Discussion

HSP typically presents with a classic tetrad of symptoms: non-thrombocytopenic purpura, polyarthralgia, abdominal pain, and renal disease. These manifestations result from widespread vasculitis. Hence, there is a general consensus that classifies the diagnostic criteria of HSP, as palpable purpura in the presence of one or more of the following: diffuse abdominal pain, biopsy showing predominant IgA deposition, arthritis, and renal involvement. The purpura, a key feature, appears as palpable lesions 2-10 mm in diameter, often in clusters, and may include ecchymoses and petechiae. These lesions are non-blanching due to blood extravasation and usually persist for several weeks, predominantly appearing on the lower legs, buttocks, and arms. They may follow a maculopapular or urticarial rash, which can mimic other conditions. Local angioedema, often affecting the knees, ankles, and hands, may precede the purpura. Polyarthralgia commonly causes weight-bearing avoidance, particularly affecting the knees and ankles, but typically resolves without lasting damage. Abdominal pain, usually diffuse and colicky, develops within a week of rash onset and may be accompanied by nausea, vomiting, and either bloody or mucoid diarrhea. Renal involvement, if present, often manifests as mild glomerulonephritis with proteinuria, microscopic hematuria, and occasionally red blood cell casts [3].

HSP is characterized by the deposition of IgA-containing immune complexes in tissues. IgA, which plays a crucial role in mucosal immunity, exists in two subclasses: IgA1 and IgA2. Only IgA1, with its unique hinge region and multiple glycosylation sites, is implicated in HSP [4]. While aberrant glycosylation is linked to the disease, the exact pathogenesis remains unclear. IgA activates complement via the alternative pathway, causing endothelial damage and widespread vasculitis. HSP often follows an unnoticed upper respiratory infection and is more common in winter and autumn. Environmental and infectious agents such as group A β-hemolytic *Streptococcus*, *Mycoplasma pneumoniae*, varicella, hepatitis B virus, food allergens, drugs, insect bites, and cold exposure have been associated with HSP. The interaction between leukocytes and endothelial cells contributes to its pathogenesis, and genetic susceptibility, including associations with HLA-DRB1*01, plays a role [5].

Diagnosis of HSP is primarily clinical, based on the presence of purpura and other symptoms of the tetrad. There are no specific laboratory tests for HSP, but tests can help exclude other conditions and rule out other systems affection. Complete blood counts are usually normal or show mild leukocytosis. Serum IgA levels may be elevated but do not correlate with disease severity. Erythrocyte sedimentation rates can be elevated. Throat swabs for group A β-hemolytic *Streptococcus* might be positive if a preceding infection is present. von Willebrand factor levels may be high due to endothelial damage. Serologic tests for autoantibodies and complement levels are generally normal. Urinalysis is crucial, often revealing microscopic hematuria and red blood cell casts [6]. A skin biopsy confirming leukocytoclastic vasculitis and IgA deposition can definitively diagnose HSP. Direct immunofluorescent studies may also show C3 and fibrinogen deposits in vessel walls [7].

Sepsis, clotting disorders, thrombocytopenia, child abuse, medication reactions, bone marrow failure syndromes, and other vasculitic conditions should be considered in the presence of purpuric rash. Clinical presentation and hematological investigations are essential for distinguishing these conditions [8].

Treatment depends on the severity and organ involvement. Most cases resolve spontaneously within 4-6 weeks, with the primary goal being supportive care and reassurance. Serious complications, such as hemorrhagic involvement of various organs, may require hospitalization and close monitoring. Joint pain and soft tissue edema can be managed with NSAIDs, mostly acetaminophen, while corticosteroids may be needed for the rapid resolution of symptoms or in cases of significant abdominal pain or scrotal involvement. Severe renal cases may require high-dose corticosteroids or other immunosuppressive treatments [9].

The prognosis for HSP is generally good, with most cases resolving fully. However, symptoms may recur, often in a milder form. Long-term outcomes are better in patients with only mild renal involvement. Persistent renal issues, such as chronic nephritis, can lead to significant long-term complications, including renal insufficiency. Gastrointestinal involvement can cause short-term morbidity, with intussusception occurring in 2-4% of cases [10]. Rare but serious complications include bowel ischemia, necrosis, and perforation. Central nervous system involvement may include encephalopathy, seizures, and other severe symptoms. Scrotal involvement can lead to orchitis or testicular torsion, potentially resulting in infertility. Hence, red flags to watch for include severe abdominal pain, especially when accompanied by gastrointestinal bleeding, signs of kidney involvement such as hematuria or proteinuria, and persistent or worsening symptoms, particularly if accompanied by systemic symptoms like fever or significant discomfort. The follow-up schedule for HSP typically starts with a visit within a few weeks of diagnosis to assess the progression and manage symptoms. Initial follow-up visits are often scheduled every 1-2 months to monitor for worsening symptoms or complications, particularly related to the skin, joints, and gastrointestinal or renal systems. For those with significant renal involvement or severe symptoms, longer-term monitoring may be required every 3-6 months. Regular urine tests and blood work are crucial to track kidney function and overall health. The frequency and nature of follow-ups should be adjusted based on individual responses to treatment and the presence of any ongoing or new symptoms [11]. 

## Conclusions

HSP is an acute, self-limiting multisystem vasculitis primarily affecting children. It impacts various organ systems, with a hallmark of palpable purpuric rash present in all cases. Management typically involves symptomatic treatment and, in some cases, immunosuppressive or immunomodulatory therapies. Although the use of corticosteroids remains debated, research indicates that they may be beneficial for patients with severe renal involvement, significant abdominal pain, and soft tissue edema. While most cases resolve on their own, careful monitoring for rare but serious complications is essential. Accurate diagnosis, effective treatment, and thorough patient education are crucial for managing symptoms and preventing severe outcomes.
